# Hydrogeochemical conceptualization of granitic-urban area for sustainable water resource management: a case study of Daejeon, Korea

**DOI:** 10.1007/s10653-025-02771-8

**Published:** 2025-10-04

**Authors:** Hye-Na Ko, Jaeyeon Kim, Kang-Kun Lee

**Affiliations:** School of Earth and Environmental Sciences, Seoul National University, 1, Gwanak-Ro, Gwanak-Gu, Seoul, Republic of Korea

**Keywords:** Hydrogeochemistry, Multivariate statistical analysis, Geochemical modeling, Conceptual model, Groundwater management, Granitic-bedrock aquifer

## Abstract

Groundwater is increasingly vital under growing demand and climate pressures, making its effective management essential for sustainable use. A thorough understanding of hydrogeochemical processes is therefore critical to secure water quality and guide resource development. This study develops a conceptual model of a granitic aquifer in Daejeon, Korea, representing a typical weathered-fractured system under mixed urban and green land-use conditions. An integrated approach was applied, combining conventional geochemical analysis, multivariate statistics, geochemical modeling, and strontium isotope tracing. The results highlight silicate weathering as the dominant control on groundwater chemistry, validated by ^87^Sr/^86^Sr ratios (~ 0.716). Mineral–water interactions explain nearly half of the observed variance, mainly through the weathering of silicate minerals to secondary clays, which promote ion exchange processes. Anthropogenic activities, particularly agriculture and land use, account for ~ 15% of the variation, indicating localized contamination risks in the lowland areas. The conceptual model, supported by natural tracers, demonstrates that groundwater evolves from a Ca–HCO_3_ type in recharge zones to mixed types, such as Ca(Na)–HCO_3_ and Ca–Cl, along downgradient flow paths. This hydrogeochemical evolution reflects the combined effects of progressive mineral weathering and superimposed anthropogenic influences.

By capturing both natural processes and human impacts, this study advances the understanding of hydrogeochemical dynamics in granite-based aquifers. The proposed conceptual framework provides a basis for predicting groundwater evolution and emphasizes the urgent need for sustainable management of these vulnerable resources in rapidly urbanizing regions.

## Introduction

Groundwater is one of the most essential water resources available for continuous use when managed properly, and its significance is increasing in response to climate change, including more frequent floods and droughts and human activities (Dao et al., 2024; Famiglietti, 2014; Jasechko et al., 2024; Kuang et al., 2024). The sustainable development of groundwater resources requires an understanding of their origin and renewability (Morán-Ramírez et al., 2016). Therefore, evaluating the processes that influence the chemical characteristics of groundwater is essential for effective water resource management and protection. The chemical composition, or quality, of groundwater is a critical factor in determining its suitability for domestic, industrial, and agricultural uses (Edmunds et al., 2002; Esteller et al., 2012). In Korea, the Groundwater Act (Act No. 20231) provides the legal framework for groundwater management, assigning responsibilities to central and local governments to secure and manage resources. Nevertheless, rapid urbanization and land-use change continue to increase contamination risks, highlighting the importance of hydrogeochemical investigations.

Groundwater quality is influenced by diverse factors, including precipitation, aquifer mineralogy, geological structure, and anthropogenic pressures such as fertilizer application. Hydrogeochemistry provides valuable insights into these systems by identifying the origin of chemical constituents, estimating transit times, delineating flow paths, and characterizing water–rock interactions within aquifers (Morán-Ramírez et al., 2016). To capture such complexity, numerous studies have combined hydrochemical and geological perspectives (Hosono et al., 2020; Luo et al., 2018; Martínez & Bocanegra, 2002; Taucare et al., 2020; Wu et al., 2020).

Systematic approaches have included ionic ratios (Subba Rao et al., 2022; Zaidi et al., 2015; Zakaria et al., 2021), multivariate statistical techniques (Mohamed et al., 2022; Pathak & Limaye, 2011; Sahu et al., 2018; Sikakwe et al., 2020), and geochemical modeling (Chen et al., 2021; Marghade et al., 2021a, 2021b; Pérez-Ceballos et al., 2021; Roy et al., 2020; Subba Rao et al., 2017). Mass transfer simulations using NETPATH (Plummer et al., 1994) and PHREEQC (Parkhurst & Appelo, 2013) are particularly notable for quantifying mineral reactions. Hydrogeological works on intrusive rock aquifers, especially granites, have emphasized the importance of weathering profiles, fractures, and land-use pressures on groundwater occurrence and chemistry (Dewandel et al., 2011; Lachassagne et al., 2021; Lancia et al., 2020; Hwang et al., 2024). In Korea, groundwater characterization studies have also been conducted, but most have focused on rural and coastal areas (Kim et al., 2019; Kim et al., 2005; Chae et al., 2004; Kwon et al., 2021). Given that crystalline rocks, including granites, constitute more than half of the nation’s geology (NGII, 2017) and play a critical role in groundwater occurrence, their hydrogeochemical characterization is of particular national importance.

The rapid expansion of urban areas and economic growth, in Korea and worldwide, has further increased the demand for groundwater (Lee et al., 2007; Li et al., 2010; Flörke et al., 2018). In this context, understanding hydrogeochemical processes in regions where urban and natural land uses coexist is essential for sustainable groundwater management. This study addresses these needs by focusing on Daejeon City, a representative granitic aquifer system where industrial, residential, and forested zones overlap. By integrating geochemical analyses, multivariate statistics, geochemical modeling, and strontium isotope tracing, we develop and validate a conceptual model that captures both natural processes and anthropogenic impacts in a weathered granite aquifer. The novelty of this work lies in applying multiple analytical perspectives with isotopic validation at a regional scale, offering a more robust interpretation than earlier studies based on single approaches.

Therefore, the objectives of this study are twofold: (1) to evaluate the hydrogeochemical characteristics of groundwater in a representative granite aquifer of Korea where urban and green areas coexist, and (2) to construct a conceptual model that explains groundwater evolution under complex land-use conditions. By adopting a systematic and multi-perspective approach, this study advances the understanding of hydrogeochemical dynamics in granite-based aquifers and provides a framework for sustainable groundwater management in rapidly urbanizing regions.

## Study area and materials

### Daejeon city

**Fig. 1:**
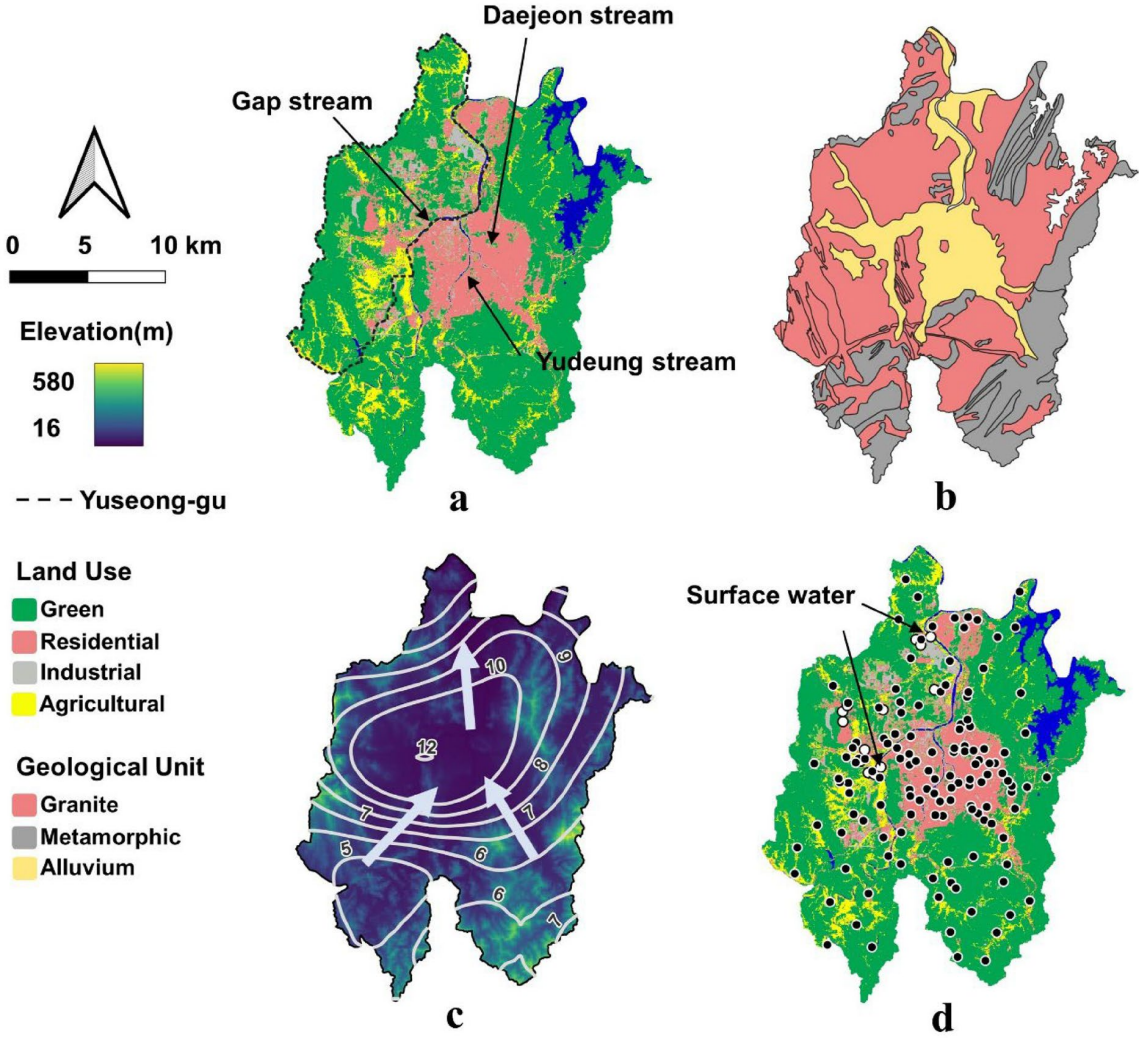
Description of the study area; **a** describes land use map of Daejeon city, South Korea, **b** shows the geological condition (black solid line represents geological divide), **c** shows the elevation (m, a.s.l.), the depth to groundwater (m, b.g.l.) and flow direction of groundwater in the Daejeon city, and **d** shows sampling points (location of wells for groundwater and surface water sampling point). Empty dots denote additional samples and 2 points for surface water sampling

The study area is located in Daejeon City, South Korea (Fig. 1a). According to geological surveys, the region is primarily composed of Jurassic plutonic rocks, including two-mica granite, biotite granite, and schistose granite (Park et al., 1977; Lee et al., 1980) (Fig. 1b). These plutonic rocks have intruded into Precambrian or Paleozoic metamorphic rocks of the Ogcheon Belt, which also contains partially metamorphosed sedimentary deposits (Hwang, 2013). In the southern portion of the study area, quartz porphyry and aplite dykes further intrude the Jurassic granite. Weathered zones of granite are extensively developed, with thicknesses ranging from 20 to 50 m (Lim et al., 1982). According to the Hydrogeological Information Map of the Geum River Basin (KIGAM, 2021), the aquifer system in this area is generally categorized as a single granite aquifer. However, the weathered regolith forms a porous aquifer that overlies the fractured granite bedrock, and together they constitute a composite hydrogeological system controlling groundwater occurrence and flow.

Over the past decade, the mean annual temperature of the study area has been approximately 13.2 °C, and the average annual precipitation has been around 1300 mm (Korea Meteorological Administration, www.kma.go.kr). Both temperature and precipitation peak sharply in August, corresponding with Korea’s monsoon season. The topography is characterized by highlands located in the northwestern and southeastern regions, with elevations ranging from 300 to 573 m above sea level (Fig. 1c). Three major urban rivers (Yudeung, Daejeon, and Gap streams) flow through the central part of the city. Groundwater generally flows from the higher elevations toward these streams, which are situated on alluvial deposits.

Land use in the study area comprises approximately 58% forest, 22% agricultural land, and 20% urban areas, including both industrial and residential zones. This diversity in land use provides a suitable setting for investigating how both natural and anthropogenic factors influence groundwater quality (Fig. 1a).

### Data sampling

Groundwater samples were collected from 131 wells distributed throughout the study area, located at various sites such as schools, parks, apartment complexes, and industrial zones, and serving different purposes. In addition, three rounds of sampling were conducted in 2021 from eight newly installed wells designated as emergency water supply facilities in Yuseong-gu (Dotted line) (Fig. 1a). The locations of all wells are shown in Fig. 1d, with the eight additional wells marked in red. For comparison, a surface water sample was also collected from the Gap Stream. Temperature, electrical conductivity (EC), total dissolved solids (TDS), and pH were measured on-site using a YSI ProDSS digital sampling system (Xylem, USA). All water samples were collected in 2-L polyethylene bottles after purging stagnant water for at least 15 min. A YSI ProDSS digital sampling system was installed in the field to monitor parameters such as temperature and dissolved oxygen, and sampling was conducted only after these parameters stabilized, ensuring representative and stable water chemistry. The collected samples were filtered through 0.45-μm membranes and refrigerated until laboratory analysis. Major cations and anions were analyzed using ion chromatography (ICS-5000; Thermo Scientific Dionex, USA) at the Korea Basic Science Institute (KBSI). To ensure data quality, charge balance error (CBE) was calculated for all samples, and only those with a CBE within ± 10% were retained. Although the conventional threshold is ± 5% (e.g., Freeze & Cherry, 1979), a broader criterion of ± 10% was adopted in this study to accommodate potential systematic errors associated with analytical techniques (Güler et al., 2002; Pacheco Castro et al., 2018). Strontium isotope ratios (^87^Sr/^86^Sr) were measured using a Neptune MC-ICP-MS instrument (Thermo Finnigan, Bremen, Germany) at KBSI. All procedural blanks contained negligible Sr^2^⁺, less than 1 ng. The ^87^Sr/^86^Sr ratios were normalized to 0.1194 for ^86^Sr/^88^Sr (Faure, 1986), with the mean isotopic ratio of the NBS987 standard reported as 0.710247 ± 0.00017 (2σ, n = 18).

## Methodology

### Analytical method

Multivariate statistical analysis was conducted to support the interpretation of groundwater quality evolution identified through conventional hydrogeochemical methods. Principal Component Analysis (PCA), a linear dimensionality reduction technique, was employed to reveal associations among variables by simplifying the dataset. In this study, the analysis included the major ions Ca^2^⁺, K⁺, Mg^2^⁺, Na⁺, HCO_3_⁻, Cl⁻, NO_3_⁻, and SO_4_^2^⁻. Parameters such as temperature, dissolved oxygen (DO), and pH were excluded: temperature and DO exhibited minimal variation during the study period, and pH was omitted due to its strong correlation with bicarbonate.

The Shapiro–Wilk test was applied to assess whether the selected variables followed a normal distribution. Before PCA, the data were standardized to have a mean of zero and a standard deviation of one (z-scale) to avoid misclassification due to differences in units or magnitudes across variables. PCA transforms the original set of correlated variables into a new set of uncorrelated (orthogonal) variables, known as principal components (PCs). The eigenvalues of the PCs represent the amount of variance explained by each component. The contributions of the original variables to the PCs are expressed as loadings, while the transformed data points are referred to as scores (Helena et al., 2000). To enhance interpretability, varimax rotation was applied to extract PCs with eigenvalues greater than 1, following the Kaiser criterion (Kaiser, 1958). Principal component analysis (PCA) was conducted using the *prcomp* function from the *stats* package (version 3.6.2) in R software (R Studio version 1.4.1717).

### Geochemical modeling

PHREEQC is a geochemical modeling program written in C +  + and developed by the United States Geological Survey (USGS). The program is designed to perform a wide range of geochemical calculations related to aqueous reactions. It supports multiple types of aqueous models and can carry out the following functions:

1. Speciation and saturation index (SI) calculations,
2. One-dimensional transport simulations with reversible and irreversible reactions, and Mass transfer calculations (Parkhurst & Appelo, 2013).

In this study, PHREEQC was employed to calculate activity ratios of dissolved constituents, determine the saturation indices of minerals, and simulate mass transfer along the groundwater flow path.

Groundwater chemistry is influenced by various processes, including flow, recharge, discharge, and fluid-rock interactions. As groundwater resides in the subsurface for extended periods, it increasingly interacts with surrounding minerals, with its chemical composition gradually controlled by weathering processes. The saturation index (SI) is a useful indicator for predicting the reactive mineralogy of the subsurface using only groundwater chemistry data, without the need for direct sampling or analysis of solid phases. SI also indicates the thermodynamic stability of a mineral in groundwater. It is calculated using the Eq. (1):

1 $${\text{SI}} = \log \left( {\frac{{{\text{IAP}}}}{{{\text{K}}_{{{\text{sp}}}} }}} \right)$$

where IAP (Ion Activity Product) is the product of the activities of the dissolved ion species, and Kₛₚ is the equilibrium constant of the mineral. If SI values equals to 0, it means water is equilibrium with respect to mineral. When SI is greater than 0, water is saturated and mineral can be precipitated into the water. When SI is lower than 0, water is unsaturated and mineral can be dissolved into the water. Inverse modeling is one of the most widely used simulation methods in hydrogeochemical research. It is particularly effective for evaluating the evolution of groundwater chemistry by simulating the chemical reactions that occur along the flow path. This approach allows for quantitative assessment of mass transfer processes from recharge to discharge zones. The mass balance of conceptual models used in inverse modeling can be expressed by the following Eq. (2):

2 $$\mathop \sum \limits_{j = i}^{n} a_{ij} x_{j} = b_{i}$$

where *a*_*ij*_ is the stoichiometric number of element *i* in mineral *j*, *x*_*j*_ is the molar number of specific minerals of gases that dissolved or precipitated, and *b*_*i*_ is increment of element *i* in the final solution compared with the initial solution (Li et al., 2010). Positive values indicate dissolution of the corresponding phase, while negative values indicate precipitation. Based on the simulation results, PHREEQC enables quantification of the key geochemical processes responsible for the observed changes in groundwater composition between two points along the flow path (Plummer et al., 1990, 1994).

## Results and discussion

### General characteristics

**Fig. 2:**
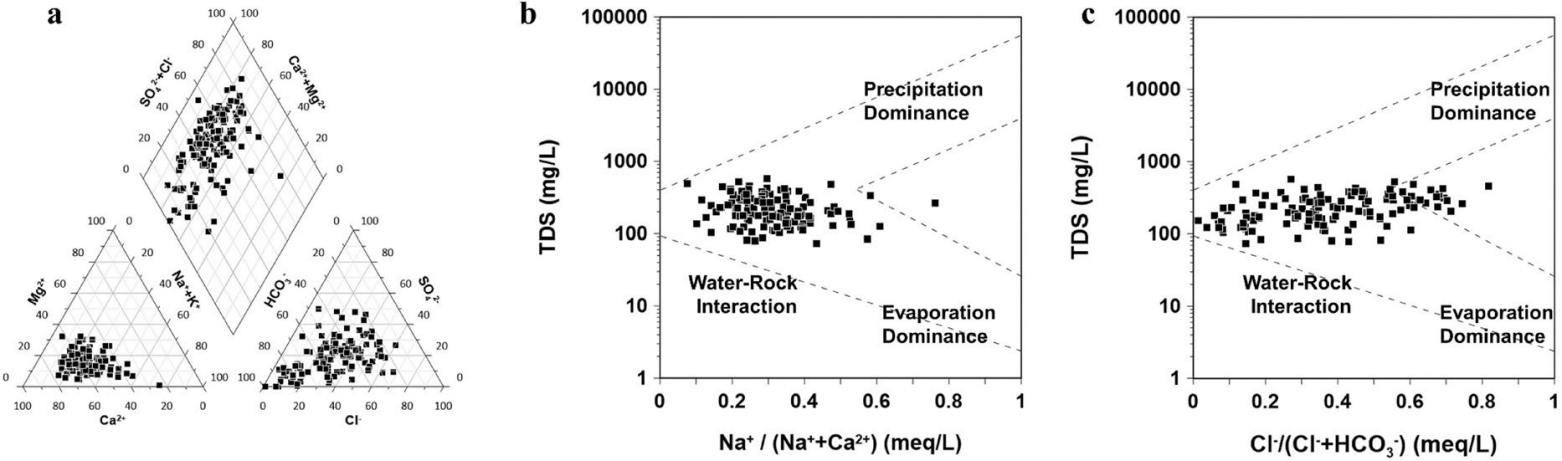
**a** Piper plot that depicts the geochemical face (water type) of the groundwater in the study area, **b** and **c** show the mechanism of controlling groundwater chemistry in the study area

The statistical summary of the physicochemical parameters of the collected groundwater samples is presented in Table 1. The pH of groundwater ranged from 5.9 to 8.6, with a small standard deviation, indicating slightly acidic to weakly alkaline conditions. These values fall within the typical range observed in groundwater from granitic basement rocks (Lee et al., 2019). Electrical conductivity (EC) ranged from 77.6 to 883 μS/cm, with an average of 323.8 μS/cm, reflecting generally freshwater quality. Total dissolved solids (TDS) concentrations varied from 73.4 to 569.6 mg/L, with a mean of 106.3 mg/L, indicating low to moderate mineralization levels. Among the dissolved ions in groundwater, Ca^2+^ and HCO_3_^−^ were the most abundant species, and major ions were in the following order: Ca^2+^  > Na^+^  > Mg^2+^  > K^+^ for cation, and HCO_3_^−^ > Cl^−^ > SO_4_^2−^ > NO_3_⁻ for anion. These results indicate that the groundwater in the study area is primarily of the Ca-HCO_3_ type, with some variations observed (Fig. 2a). Water types ranged from Ca-Na-HCO_3_ to Ca–Cl. Such facies are commonly found in fractured bedrock aquifers in Korea and suggest ongoing geochemical interactions or mixing between water bodies with different chemical compositions (Kim et al., 2014).

**Table 1:** Statistical summary of hydrogeochemistry data of sampled groundwater in the study area

Variables	Max.^a^	Min.^b^	Mean	SD^c^
(n^d^ = 131)				
T^e^(℃)	22.9	13.6	16.8	1.5
pH	8.6	5.9	6.7	0.5
EC^f^ (μs/cm)	883.0	77.6	323.8	173.0
TDS^g^(mg/L)	30.3	0.0	6.6	6.1
Ca^2+^ (mg/L)	115.0	6.6	39.9	22.3
Mg^2+^ (mg/L)	28.8	0.5	6.3	4.3
Na^+^ (mg/L)	71.2	3.5	19.2	11.0
K^+^ (mg/L)	11.2	0.4	2.0	1.7
HCO_3_^−^ (mg/L)	326.5	12.2	90.0	52.4
Cl^−^ (mg/L)	104.0	1.1	31.5	24.9
SO_4_^2−^ (mg/L)	186.9	0.1	30.1	27.3
NO_3_^−^ (mg/L)	134.0	0.0	29.0	26.8
SiO_2_ (mg/L)	25.5	2.4	14.7	5.0

^a^Max: Maximum

^b^Min: Minimum

^c^SD: Standard Deviation

^d^n: number of samples

^e^T: Temperature

^f^EC: Electrical Conductivity

^g^TDS: Total Dissolved Solid

The Ca-Na-HCO_3_ type usually reflects natural water quality in aquifer systems, formed by the enrichment of Na^+^ through water–rock interactions, such as the weathering of silicate minerals or ion exchange processes (Chae et al., 2006, 2007). Groundwater with a Ca–Cl type indicates contamination from anthropogenic sources, such as the application of chemical fertilizers and sewage, leading to increased levels of Cl^−^, SO_4_^2−^, and NO_3_^−^ in the groundwater (Hwang et al., 2017; Kumar, 2014; Prasanna et al., 2010).

Figure 2b and c illustrate the mechanisms controlling the groundwater chemistry in the study area using Gibbs plots (Gibbs, 1970), which relate TDS to the ionic ratios Na^+^/(Na^+^ + Ca^2+^) and Cl^−^/(Cl^−^ + HCO_3_^−^). The plot is divided into three mechanisms: evaporation, precipitation, and water–rock interaction domains. All samples fall within the water–rock interaction domain, indicating that the interactions between groundwater and the aquifer matrix are the primary processes controlling the chemical properties of groundwater in this study area. The study area comprises thick alluvial deposits that are widely distributed, as well as the underlying highly weathered granitic bedrock. Such geological conditions enhance porosity and hydraulic conductivity, thereby creating varied hydrogeological settings that may significantly influence groundwater chemistry. Previous studies in the Daejeon area have indeed shown that groundwater chemistry varies with well depth (4-440 m), with shallow wells (0-30 m) reflecting surface-derived contaminants (e.g., NO_3_⁻, K⁺), intermediate depths (60-90 m) showing strong water–rock interaction signals (e.g., Na⁺, Ca^2^⁺, Cl⁻), and deeper wells (up to 90 m) characterized by elevated F⁻ concentrations due to prolonged mineral dissolution (Jeong 2001a, b).

### Hydrogeochemical process

According to Sect. "Analytical method", the primary process controlling groundwater quality is the interaction between groundwater and the aquifer matrix. This interaction plays a crucial role in shaping groundwater chemistry and serves as a valuable tool for understanding the genesis of groundwater quality. Ca^2+^ and Na^+^ for cations, and HCO_3_^−^ and Cl^−^ for anions are key components that determine water type in this study area. To distinguish the types of weathering processes based on geological conditions, the concentration ratios of dissolved ions were examined using bivariate plots.

**Fig. 3:**
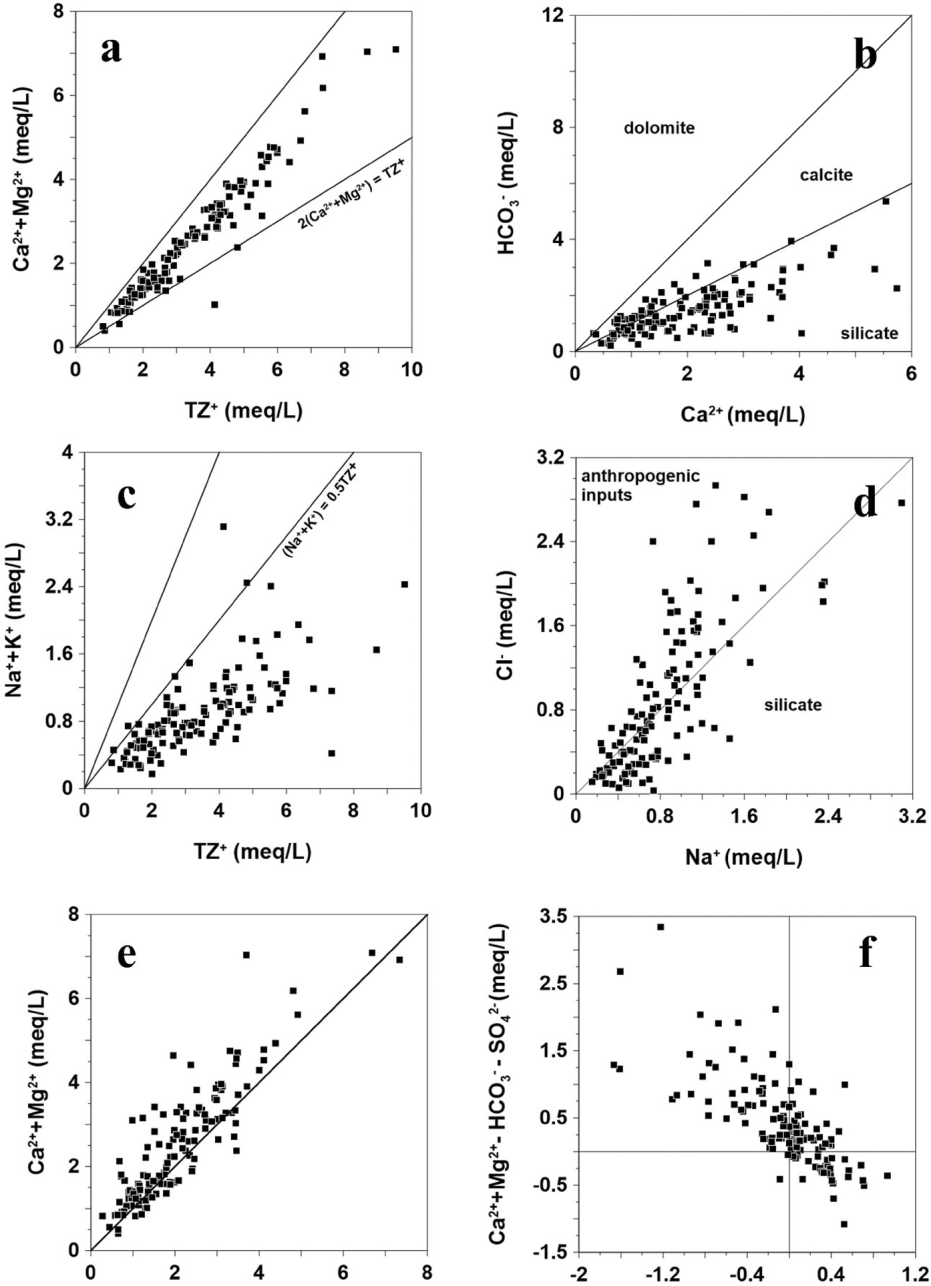
Ion bivariate plot of groundwater in the study area

The plot of (Ca^2+^ + Mg^2+^) versus total cations (TZ^+^ = Ca^2+^ + Mg^2+^ + Na^+^ + K^+^ in meq/L) (Fig. 3a) shows that most samples lie below the 1:1 line, indicating a substantial contribution of alkali metal ions (Na⁺ + K⁺) to the total cation concentration (Kumar et al., 2009). The depleted balance is likely made up by other cations or alkali metal ions. The equivalent ratio between Ca^2+^ and HCO_3_^−^ is 1:2 and 1:4 in silicate terrain (Fig. 3b), assuming that Ca^2^⁺ and HCO_3_⁻ are derived exclusively from the dissolution of calcite and dolomite, respectively (Holland, 1978; Veizer & Mackenzie, 2003). Most samples fall within the silicate weathering domain, while some fall within the calcite dissolution zone. According to the geological map (Fig. 1a), carbonate minerals are scarce in the granitic bedrock, where calcite (if present) typically occurs as a fracture-filling mineral (Kim et al., 2001). However, due to the significantly higher dissolution rate of calcite (approximately 120 times faster than that of silicates), even a small amount can considerably affect the concentrations of Ca^2^⁺ and HCO_3_⁻ in groundwater (Meybeck, 1987).

According to the relation between TZ^+^ and concentration of alkali metal ions, most of the samples are plotted below the Na^+^ + K^+^ = 0.5TZ^+^ line (Fig. 3c). This suggests that silicate mineral weathering and/or soil salts significantly contribute to the supply of alkali metal ions in groundwater in this study area. Figure 3d, showing Na^+^ versus Cl^−^ in water samples, reveals that some samples are plotted below the equivalent line. This indicates an excessive Na^+^ compared to Cl^−^, implying that the weathering of Na-bearing silicates, such as albite, is the main source of the excessive Na^+^. In contrast, samples plotting above the line indicate an excess of Cl⁻. As no geological evidence suggests the presence of halite in the study area, the Cl⁻ is unlikely to originate from geologic sources. Instead, typical anthropogenic sources of Cl⁻ include animal waste, chemical fertilizers, domestic sewage, and landfill leachate (Nagarajan et al., 2010; Tay, 2021).

Figure 3e shows a bivariate plot of the sum of alkaline earth metal ions concentration versus HCO_3_^−^and SO_4_^2−^. According to the plot, most of the samples are plotted above the equivalent line, meaning that large amounts of Ca^2+^ or Mg^2+^ can be derived from non-carbonate sources, and excessive positive charge by those ions might be balanced by other anions like Cl^−^ from anthropogenic inputs (Srinivasamoorthy et al., 2008; Varol & Davraz, 2014). Moreover, the dominance of Ca^2+^ and Mg^2+^ over HCO_3_^−^ and SO_4_^2−^ may also result from ion exchange, which is defined by the process that alkali metal ions like Na^+^ and K^+^ in groundwater might be exchanged with Ca^2+^ or Mg^2+^ from aquifer matrix such as clay minerals. The relation between (Ca^2+^ + Mg^2+^-HCO_3_-SO_4_^2−^) versus (Na^+^-Cl^−^) also supports the ion exchange process inside the aquifer. If the ion exchange process is the dominant process in the system, then the sample should form a linear relation between (Ca^2+^ + Mg^2+^-HCO_3_-SO_4_^2−^) and (Na^+^-Cl^−^) with a slope of -1 (Rajmohan & Elango, 2004). The linear relation between the two variables from Fig. 3f is -1.02, which strongly supports the hypothesis that ion exchange significantly influences the groundwater chemistry in the study area.

Lastly, a thermodynamic calculation using the geochemical modeling code PHREEQC was conducted. First, mineral stability diagrams delimiting the most stable silicate phases in the fluid were used to study the weathering process of silicate minerals (Garrels, 1965). In particular, silicate minerals, which constitute most of the bedrock in this study area, tend to undergo incongruent dissolution, retaining part of the released silica through the formation of authigenic minerals. In contrast, other minerals like carbonates experience congruent dissolution (Appelo & Postma, 1993; Walraevens et al., 2018). The activity ratios of Na⁺, K⁺, Ca^2^⁺, and H_4_SiO_4_ were calculated using the geochemical modeling software PHREEQC (Parkhurst & Appelo, 2013).

**Fig. 4:**
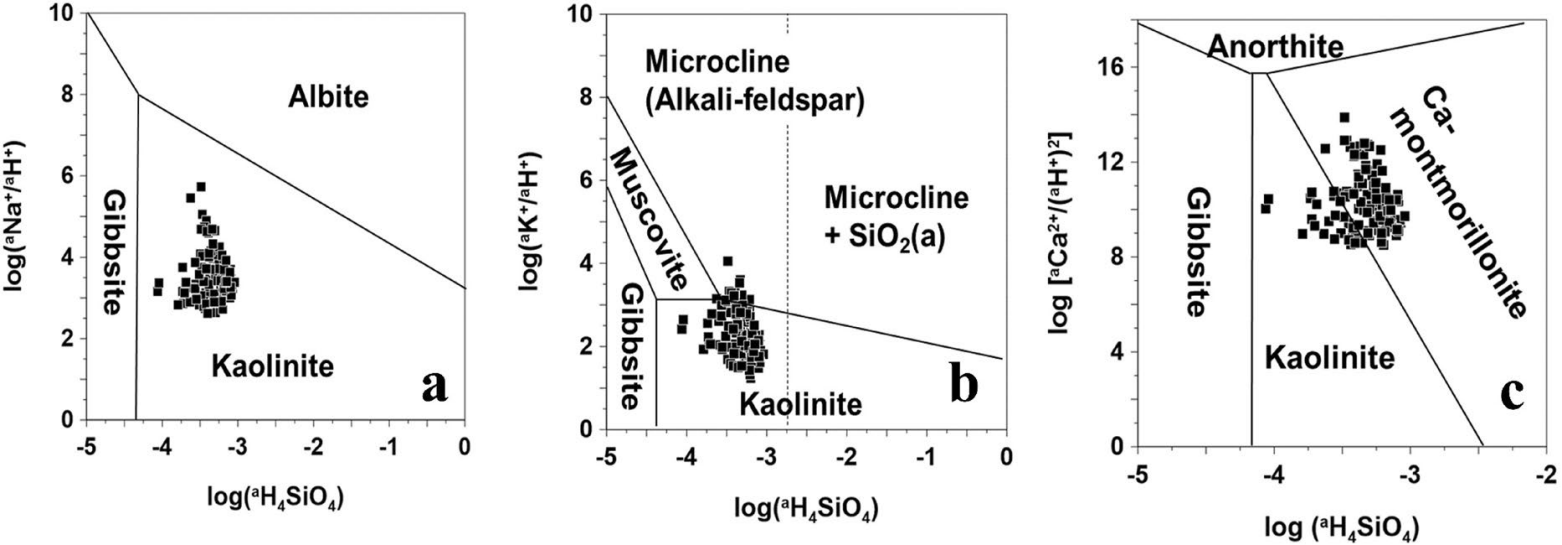
Stability diagram of silicate minerals: **a** Na-system, **b** K-system, and **c** Ca-system

Figure 4 shows the stability diagram for the Na-, K-, and Ca-systems. According to the diagram, most of the samples are plotted in the kaolinite and montmorillonite fields, with a few lying along the boundary between the K-feldspar, muscovite, and microcline fields. The equilibrium of most groundwater samples with kaolinite is consistent with previous findings by Freeze and Cherry (1979), who reported that the weathering of feldspar and biotite into kaolinite is a typical reaction pathway in granitic bedrock aquifers (Walraevens et al., 2018). Notably, none of the samples fall within the albite or anorthite fields, suggesting that these minerals are not in equilibrium with the sampled groundwater and are therefore prone to dissolution. This suggests that these silicates undergo chemical weathering, releasing cations and transforming into stable clay minerals like kaolinite. The weathering of feldspars appears to be a crucial natural factor influencing the hydrochemical evolution of groundwater in the study area.

**Fig. 5:**
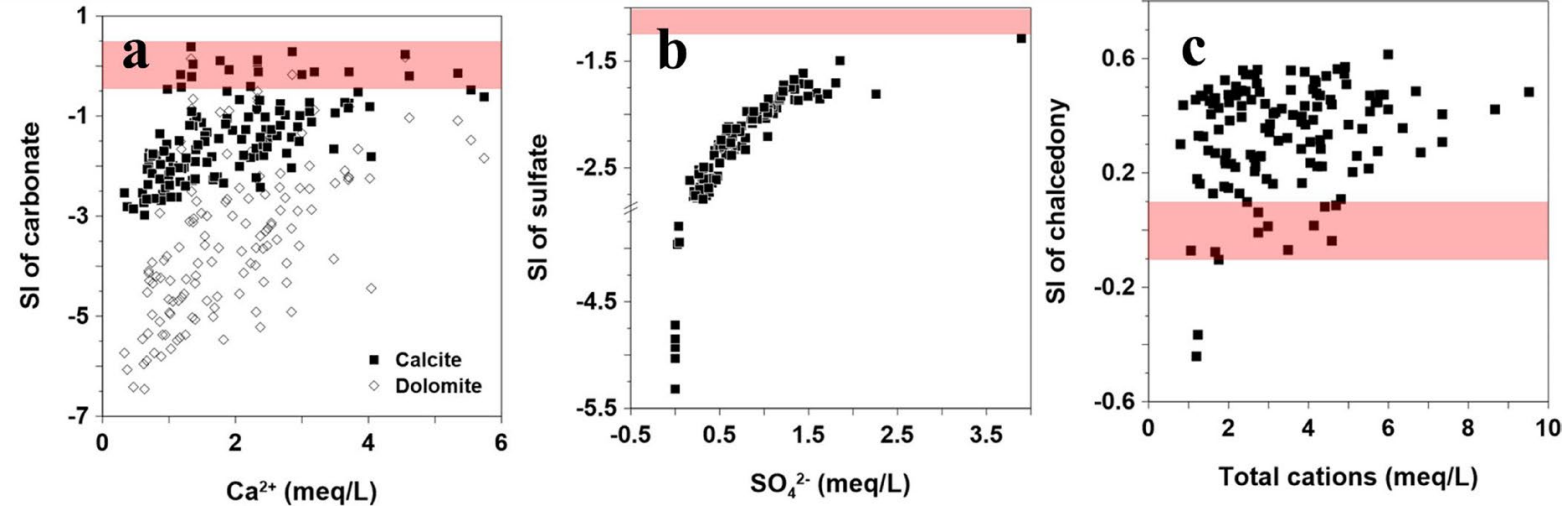
Saturation indices (SI) of selected minerals: **a** carbonate, **b** gypsum, and **c** chalcedony

Saturation indices (SIs) of other minerals were also calculated using PHREEQC. Carbonate and sulfate minerals undergo congruent dissolution, releasing major ions such as Ca^2^⁺, HCO_3_⁻, and SO_4_^2^⁻. According to previous studies, the SI of a specific mineral is positively correlated with the concentrations of its related components (e.g., the dissolution of calcite increases the concentrations of Ca^2^⁺ and HCO_3_⁻). Therefore, the dissolution of these minerals plays a key role in the increase of dissolved ions (Wu et al., 2021). In this study, the SIs of calcite, dolomite, gypsum, and chalcedony were calculated. The relationship between the related ions and their corresponding SIs was illustrated using bivariate plots. The red-colored sections in each graph indicate the conditions under which the minerals are in equilibrium (Fig. 5).

Figure 5a shows the relationship between Ca^2^⁺ concentration and the SIs of calcite and dolomite. The majority of the samples exhibit negative SI values, indicating that the dissolution of calcite and dolomite contributes to the increase in Ca^2^⁺ concentration in groundwater. However, there is limited geological presence of dolomite in the study area. Therefore, calcite, which occurs as a fracture-filling mineral and as a major component of sedimentary deposits, is likely the primary source of Ca^2^⁺ and HCO_3_⁻. Additionally, the continuous release of Ca^2^⁺ from Ca-bearing silicate minerals could promote calcite precipitation, thereby increasing the SI of calcite. This is supported by the observation that some calcite SIs are close to equilibrium.

Figure 5b illustrates the relationship between the SI of gypsum and SO_4_^2^⁻ concentration. Similar to the behavior of carbonates, most gypsum SIs indicate undersaturated conditions, meaning that gypsum dissolution is a reliable source of Ca^2^⁺ and SO_4_^2^⁻ in the groundwater. Other potential sources of SO_4_^2^⁻ include reactions such as the oxidation of fracture-filling minerals like pyrite (Cloutier et al., 2008; Glynn & Brown, 2011; Kim et al., 2019) and anthropogenic activities, such as the use of chemical fertilizers in agriculture (Kim et al., 2005; Liu et al., 2020; Roy et al., 2020).

Figure 5c focuses on chalcedony and its SI is commonly used as an indicator of silicate weathering (Lee et al., 2021). In this study, the SIs of most groundwater samples, with a few exceptions, were greater than zero. Moreover, the SIs increased with the sum of cation concentrations (TZ⁺), confirming that silicate hydrolysis is a key process contributing to the formation of cations in groundwater (Fig. 5c).

### Anthropogenic factor affecting groundwater quality

To identify the factors controlling water quality in the study area, principal component analysis (PCA), a type of multivariate statistical analysis, was conducted on pre-processed raw data. The analysis included major ions and physicochemical characteristics such as pH, TDS (total dissolved solids), EC (electrical conductivity), and total hardness. Table 2 presents the component loadings, eigenvalues of the matrix, and the variance explained by the principal components. Three components were extracted using the Kaiser criterion (Kaiser, 1958), explaining 76% of the total dataset. To investigate the PC scores concerning land use and site-specific characteristics, such as the coexistence of urban and forest areas, sampling wells were classified into three categories: residential/industrial, agricultural, and forest areas.

**Table 2:** PCA results for selected groundwater quality variables

	PC 1	PC 2	PC 3
pH	0.04	− 0.78	0.05
EC	0.97	0.01	0.03
TDS	0.98	0.15	− 0.06
Ca^2+^	0.94	− 0.15	− 0.04
Mg^2+^	0.8	0.01	0.18
Na^+^	0.7	0.19	− 0.1
K^+^	0.31	0.22	0.76
HCO_3_^−^	0.68	− 0.59	− 0.04
Cl^−^	0.8	0.33	− 0.03
SO_4_^2−^	0.8	0.06	0.13
NO_3_^−^	0.37	0.72	0.03
SiO_2_	0.24	0.23	− 0.83
Eigenvalue	5.95	1.78	1.33
Explained %	50	15	11

**Fig. 6:**
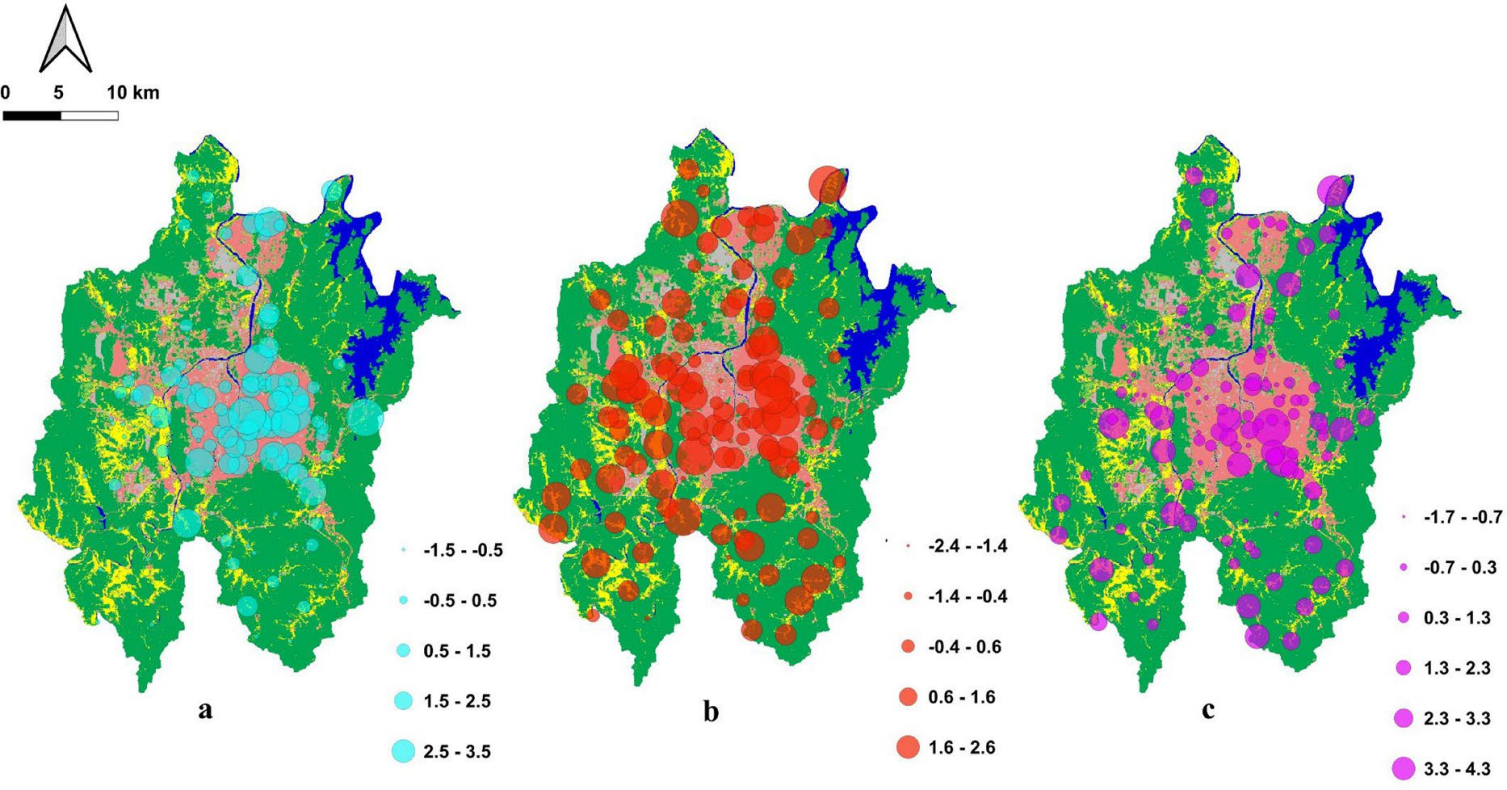
Spatial distribution of each principal component (PC) and each point is scattered on the land use map (refer to Fig. 1a); **a** PC1, **b** PC 2, and **c** PC 3

The first principal component (PC1) explains approximately 50% of the total variance. It is characterized by high positive loadings for EC, TDS, and a wide range of dissolved ions, including Ca^2^⁺, K⁺, Mg^2^⁺, Na⁺, HCO_3_⁻, Cl⁻, NO_3_⁻, and SO_4_^2^⁻. The high loadings of EC and TDS indicate that this component reflects the degree of overall mineralization (Nair et al., 2020). Additionally, Cl⁻ and SO_4_^2^⁻, which are indicators of groundwater contamination, also show high loadings, suggesting the influence of anthropogenic factors related to human activities. The spatial distribution of PC1 scores (Fig. 6a) shows higher values in residential/industrial zones at lower elevations, in contrast to lower scores in forested areas at higher elevations. This pattern implies intensified water–rock interactions and potential anthropogenic influence as groundwater moves from recharge to discharge zones. Therefore, PC 1 likely reflects a combination of natural processes, anthropogenic inputs, and mixed sources (Belkhiri & Narany, 2015; Chai et al., 2020; K. H. Kim et al., 2014; Nair et al., 2020; Nakagawa et al., 2021).

The second component (PC 2) accounts for 15% of the variance and is dominated by nitrate. High PC2 scores are concentrated in farmland and residential areas (Fig. 6b), suggesting that this component reflects nitrate contamination from anthropogenic sources, as nitrate is not derived from local geological formations. Jeong (2003) applied δ^15^N analysis to determine the sources of nitrate in groundwater in Daejeon City and identified leakages from municipal sewage pipelines and septic tanks as the primary nitrate sources. This supports the interpretation that PC 2 is strongly associated with human activities and contamination, as well as with land use patterns.

The third component (PC 3), which explains 11% of the dataset, is characterized by a negative correlation between potassium and silica ions, suggesting that these ions originate from different sources. SiO₂ in groundwater typically comes from the dissolution of silicate minerals, which reflects water–rock interactions (Dobrzyński, 2005; Njitchoua et al., 1997). If both originated from silicate weathering (e.g., biotite), a positive correlation would be expected. Instead, the inverse relationship, along with higher PC3 scores in residential/industrial areas, indicates an anthropogenic influence distinct from that represented by PC2. Potential sources of anthropogenic potassium include domestic waste, agricultural activities, and sewer infiltration (Kumar et al., 2009; Raju et al., 2015; Singh et al., 2017; Choi et al., 2005). Although potassium can be released from the weathering of feldspars and micas, this process is usually accompanied by elevated silica concentrations. In the present dataset, however, increased K⁺ levels are not associated with higher Si, suggesting that the source of K⁺ is unlikely to be solely mineral weathering. Moreover, the absence of a clear correlation with either Cl⁻ or nitrogen species complicates the attribution to road salt or sewage leakage. This pattern may indicate localized anthropogenic inputs or ion-exchange processes rather than purely natural geochemical reactions. Additionally, sampling points with high PC 3 scores are located in areas with different geological compositions compared to the other points, which are predominantly underlain by granitic bedrock. This observation suggests that local geological variability may also influence PC3, and further investigation, through additional sampling and analysis, is required to clarify the sources of potassium beyond the anthropogenic contributions already considered.

### Conceptualize the aquifer system

#### Mass transfer simulation

The hydrogeochemical characteristics, ionic ratio plots, and thermodynamic stability diagrams indicate that the chemical composition and evolution of the groundwater are influenced by interactions between groundwater and the aquifer matrix (minerals and rocks), as well as by anthropogenic factors such as nitrate and chloride. Quantitative descriptions obtained through inverse modeling simulations further support these findings and help validate the geochemical differences between groundwater types along the flow path.

Mass transfer simulation, referred to as inverse modeling, was conducted using PHREEQC (Parkhurst & Appelo, 2013). Based on the geochemical findings and geological conditions of the study site, potential mineral phases were selected for simulation, including calcite, gypsum, albite, anorthite, plagioclase, feldspar, kaolinite, Ca-montmorillonite, illite, and ion exchange. Carbon dioxide gas was assumed to be present along the groundwater flow. To minimize the influence of anthropogenic factors, nitrate was excluded from the calculations, which was reflected in the selection of the flow path. The flow path was determined based on topographic elevation, with groundwater evolving from a Ca-HCO_3_ type (initial water) to a mixed type (final water), which are the dominant water types observed in the study area. In addition, recharge predominantly occurs in the upland areas, where contamination risks are relatively low according to the land-use map, and groundwater flows downgradient toward the lowland industrial and residential zones, where anthropogenic influences are more pronounced. This interpretation is further supported by the observed increase in EC and TDS values along the flow direction, indicating progressive mineral weathering. The simulation results are presented in Table 3. The four models reported in Table 3 illustrate different mass transfer combinations concerning the precipitation or dissolution of several mineral phases, which contribute to the differences in chemical composition between the initial and final water samples.

**Table 3:** Mass transfer for inverse modeling (value in mmol/kgw). Thermodynamic database is from PHREEQC

	Model 1	Model 2	Model 3	Model 4
Phase				
CO_2_(g)	7.96E+00	4.63E+00	1.59E+01	1.59E+01
Calcite	− 7.96E+00	− 4.63E+00	− 1.59E+01	− 1.59E+01
Gypsum	6.30E−04	6.50E−04	6.30E−04	6.30E−04
Albite	0.00E+00	0.00E+00	1.07E+01	1.07E+01
Anorthite	7.96E+00	4.63E+00	1.32E+01	1.32E+01
Plagioclase	0.00E+00	0.00E+00	0.00E+00	0.00E+00
K-feldspar	5.45E−05	0.00E+00	5.45E−05	0.00E+00
Kaolinite	− 7.96E+00	− 4.63E+00	0.00E+00	0.00E+00
Ca-Montmorillonite	− 1.14E−04	0.00E+00	− 1.59E+01	− 1.59E+01
Illite	2.08E−03	2.10E−03	2.08E−03	2.08E−03
CaX_2_	2.05E−04	1.49E−04	5.33E+00	5.33E+00
MgX_2_	3.23E−04	3.26E−04	3.23E−04	3.23E−04
NaX	− 1.06E−03	− 1.01E−03	− 1.07E+01	− 1.07E+01

The simulation results indicate that the dissolution of carbon dioxide gas enhances the dissolution of minerals along the flow path. Specifically, gypsum, albite, anorthite, K-feldspar, and illite were found to dissolve during groundwater transport. Among these, the weathering of gypsum and anorthite contributes significant amounts of Ca^2^⁺, the dominant cation responsible for defining the hydrogeochemical facies in the study area. Additionally, the dissolution of albite and K-feldspar serves as a source of alkali metal ions (e.g., Na⁺, K⁺) in groundwater.

The continuous supply of Ca^2^⁺ leads to the precipitation of calcite, thereby increasing the saturation index (SI) of calcite along the flow path. This precipitation process is attributed to the common ion effect, wherein the elevated Ca^2^⁺ concentrations, primarily sourced from gypsum dissolution, promote calcite supersaturation (Jin et al., 2010; Karimi et al., 2019; Zheng et al., 2018). The dissolution of silicate minerals such as albite, anorthite, and K-feldspar occurs through incongruent weathering, releasing cations while forming kaolinite and montmorillonite, the stable clay phases confirmed in the mineral stability diagram (see Sect. 4.2). In terms of ion exchange, the simulation results indicate reverse ion exchange, resulting in an excess of alkaline earth metal ions over alkali metal ions. Therefore, geochemical evolutions from initial (Ca-HCO_3_ type) to final water (mixed type) can be summarized as:

$$\begin{aligned} {\text{Ca}} & - {\text{HCO}}_{{3}} {\text{water }} + {\text{ CO2}}\left( {\text{g}} \right) + {\text{ Gypsum }} + {\text{ Anorthite }} \\ & + {\text{ Albite }} + {\text{ K}} - {\text{feldspar }} + {\text{ Illite }} + {\text{ CaX}}_{{2}} \\ & + {\text{ MgX}}_{{2}} \to {\text{ Mixed type water }} + {\text{ Calcite }} + {\text{ Kaolinite}} \\ & + {\text{ Ca}} - {\text{montmorillonite }} + {\text{ NaX}} \\ \end{aligned}$$

#### Validation of the findings

The hydrogeochemical characteristics, ionic ratio plots, and thermodynamic stability diagrams collectively indicate that groundwater chemistry in the study area is primarily governed by water–rock interactions, with additional influence from anthropogenic sources such as nitrate and chloride. Inverse modeling simulations were conducted to further validate the geochemical differentiation of groundwater types along the flow path. An additional study using strontium isotopes (^87^Sr/^86^Sr) as natural tracers was performed to assess the influence of mineralization on groundwater chemistry. This approach adds value to the conceptual model of the aquifer system. Among natural tracers, strontium isotopes were selected because the ^87^Sr/^86^Sr ratios of minerals in igneous and metamorphic rocks are both stable and representative over geologic time (Shand et al., 2009). Moreover, the predominance of granite, which contains Sr-bearing minerals, in the study area makes strontium isotopes particularly suitable for investigating water–rock interaction processes.

**Fig. 7:**
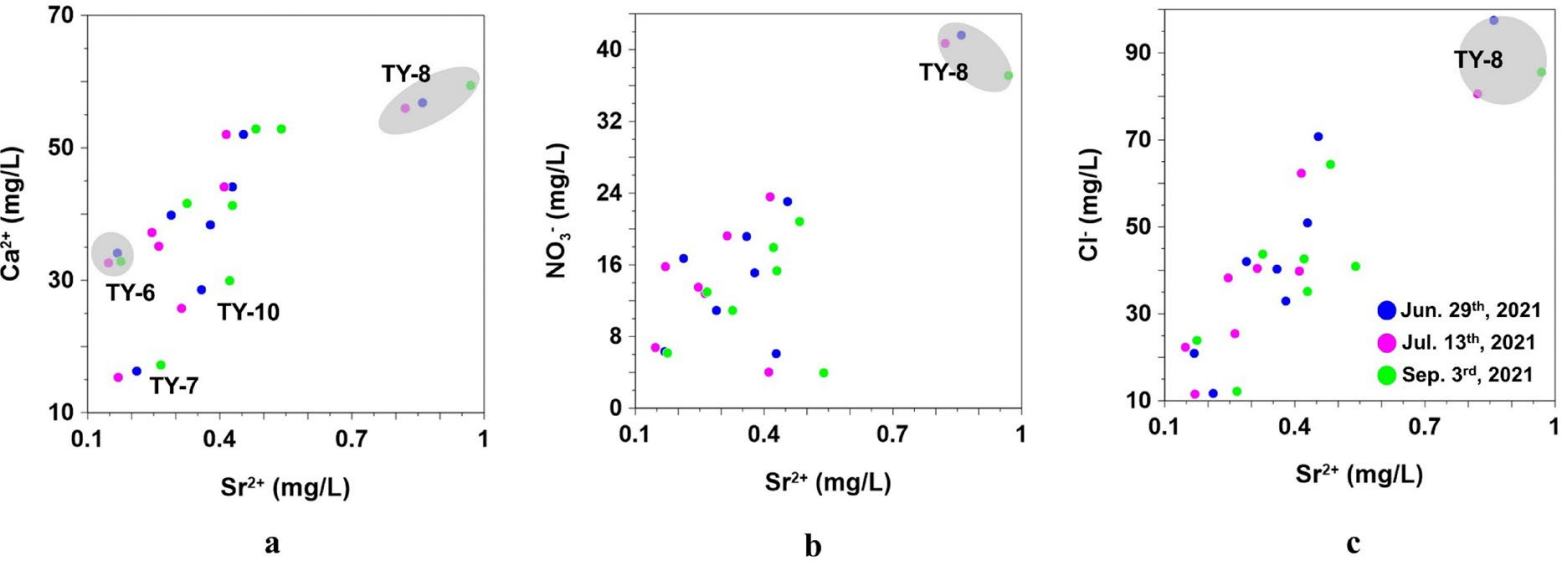
Relation between Sr^2+^, **a** Ca^2+^, **b** NO_3_^−^, and **c** Cl^−^

To minimize anthropogenic influence, eight additional samples were collected from the least-contaminated district and analyzed three times. Bivariate plots of Sr^2^⁺ versus selected species are presented in Fig. 7. Figure 7a shows the range of strontium and Ca^2+^ concentrations in additional groundwater samples analyzed for strontium isotope ratios, revealing a correlation between Ca^2^⁺ and Sr^2^⁺. This correlation indicates that Sr^2^⁺ can be used to trace the sources of Ca^2^⁺ dissolved in groundwater (Clow et al., 1997). Sample TY-7 exhibits the lowest Sr^2^⁺ concentrations than any other samples, while TY-8 had the highest (Fig. 7a). Figures 7b and 7c illustrate the relationship between Sr^2^⁺, nitrate, and Cl⁻. While nitrate is typically derived from anthropogenic sources, Cl⁻ behaves conservatively in the hydrological cycle but can also originate from human activities. Thus, a positive correlation between Sr^2^⁺ and these parameters may indicate anthropogenic contributions (Négrel & Petelet-Giraud, 2005). TY-8 exhibits enriched concentrations of Sr^2^⁺, NO_3_^−^, and Cl⁻, indicating that contamination influences groundwater chemistry beyond mineral dissolution processes. Wells with higher concentrations of nitrate and Cl⁻, including TY-8, are located near apartment complexes and industrial/research facilities, suggesting that groundwater quality may be exposed to pollutants.

**Fig. 8:**
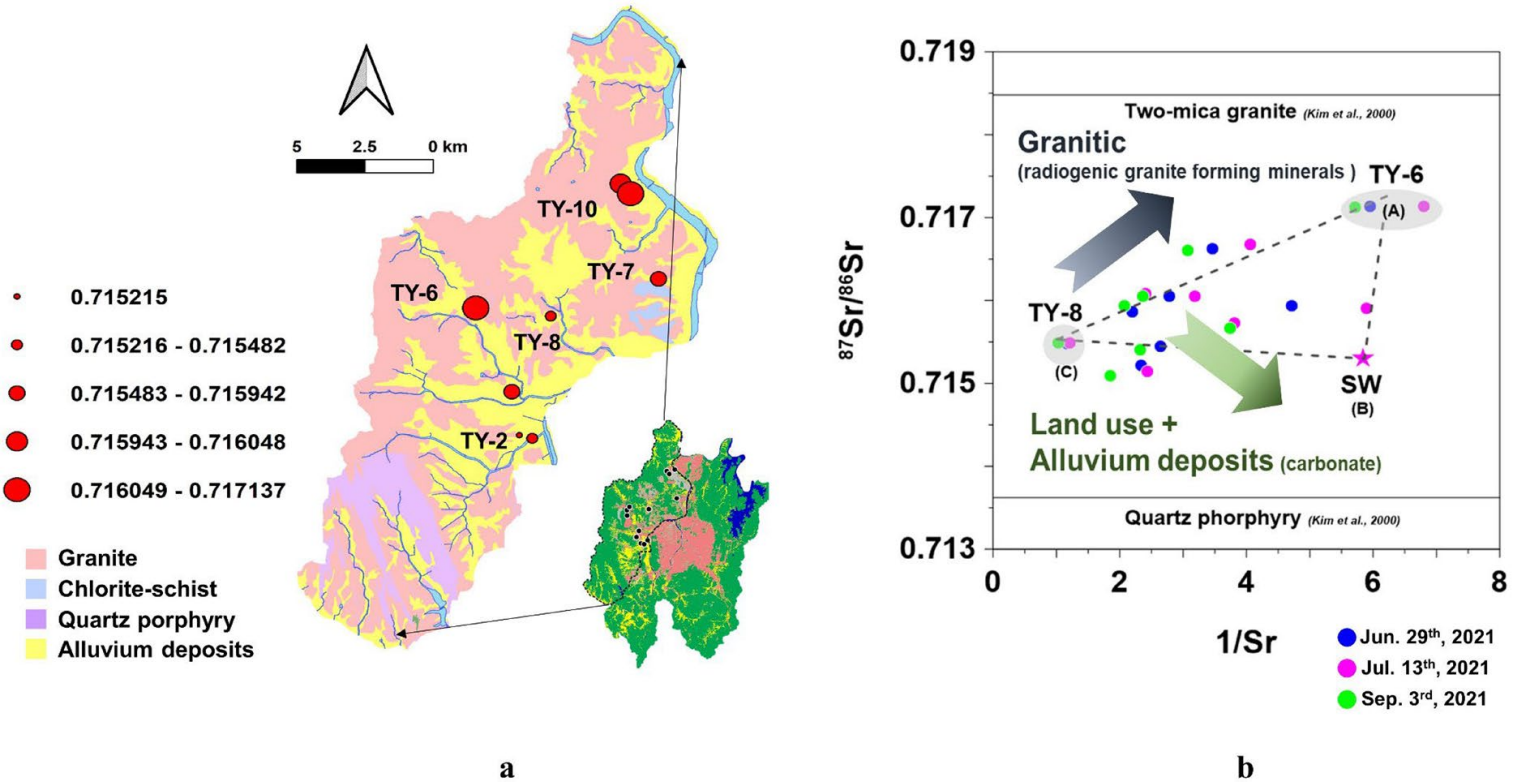
Strontium isotopic ratios: **a** spatial distribution of strontium isotope ratio with geological condition, and **b** ^87^Sr/.^86^Sr versus 1/Sr distribution in groundwater and surface water (SW)

Figure 8a depicts the spatial distribution of strontium isotopic ratios, which range from 0.71509 to 0.71714 with a mean of 0.71598 ± 0.00061, indicating minimal variation. In contrast, the isotopic ratio for surface water is 0.71530, which is less radiogenic than that of the groundwater samples, except for TY-2. According to Kim et al. (2001), the ^87^Sr/^86^Sr ratios of two-mica granite and quartz porphyry, the main rock types in Daejeon, were 0.71981 and 0.71362, respectively. The range of ^87^Sr/^86^Sr for groundwater samples falls within a similar range as those of the two rock samples, suggesting that dissolved ions such as Ca^2^⁺ and Sr^2^⁺ may originate from the dissolution of granites (Fig. 8b).

Based on the relationship between ^87^Sr/^86^Sr and 1/Sr (Fig. 8b), three end-members were identified:

1. Zone A (TY-6): high 1/Sr and the most radiogenic
2. Zone B (Surface Water): high 1/Sr but less radiogenic, reflecting dilution or surface inputs.
3. Zone C (TY-8): low 1/Sr and low radiogenic signature, strongly influenced by anthropogenic inputs.

The absence of a direct linear relationship among all samples demonstrates the presence of multiple end-members in the aquifer system (Negrel et al., 2003).

Samples plotted near zone A show more radiogenic and higher 1/Sr ratios than others, implying influence by K-bearing silicate minerals like K-feldspar and biotite (Clow et al., 1997; Santoni et al., 2016). Most groundwater samples from the alluvial and residential/industrial zones cluster in Zone B, consistent with potential contamination from surface sources. Zone B's relatively low radiogenic signature and 1/Sr values may reflect contributions from Sr-bearing, less-radiogenic minerals such as anorthite and plagioclase, or from carbonate dissolution, particularly calcite, which is frequently observed as a fracture-filling mineral in the study area (Hwang, 2013). Although the range of ^87^Sr/^86^Sr in groundwater is narrow, its spatial distribution reflects the bedrock types and provides further evidence for silicate weathering as the dominant control on groundwater chemistry, with limited influence from carbonates such as calcite. These findings are consistent with results from geochemical analyses and mass transfer simulations, thereby reinforcing the conceptual model of groundwater evolution in the Daejeon aquifer system.

### Integrated conceptual model of groundwater flow and hydrogeochemistry in a granite aquifer system

**Fig. 9:**
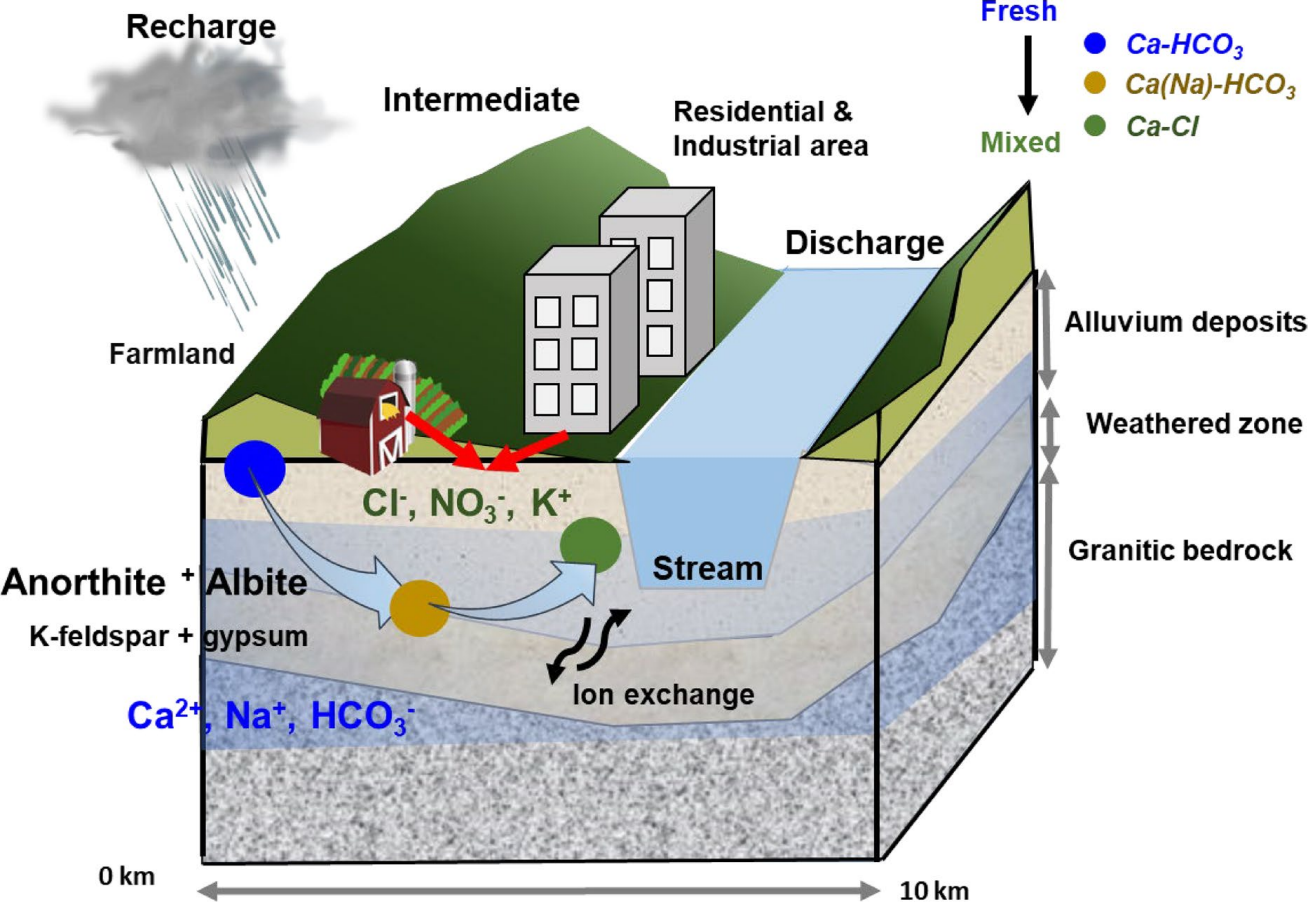
Conceptual model of the hydrogeochemical system of Daejeon city, Korea. The size of the text corresponds to the quantity of ions (or minerals, reaction) transferred

The conceptual model of the aquifer system in Daejeon City is illustrated in Fig. 9, integrating findings from mass transfer simulations, geochemical analyses of natural processes such as water–rock interactions, and anthropogenic influences. As groundwater is recharged in high-elevation areas and flows along the regional gradient, freshwater undergoes a series of geochemical reactions that result in the formation of diverse groundwater types.

Regarding the natural processes that control the geochemistry of the study area, the major cations that define the water type originate from the dissolution of silicate minerals such as anorthite, albite, and feldspars. Upon releasing cations into the groundwater, these minerals transform into more stable phases, such as clay minerals like kaolinite or montmorillonite. Additionally, the excessive Ca^2^⁺ ions supplied from the dissolution of gypsum or silicates can lead to the precipitation of calcite, resulting in an increased saturation index. Ion exchange also plays a role in the evolution of water chemistry. Consequently, recharged water evolves into a more complex mixed-type groundwater through continuous water–rock interactions. In addition, the weathered granite zone is widely distributed and highly permeable, which increases the likelihood of vertical mixing between shallow recharge and deeper groundwater. Previous studies conducted in the same region have also reported evidence of vertical mixing, particularly before and after rainfall events (Kim & Lee, 2023).

Anthropogenic factors further complicate groundwater chemistry. In particular, areas with dense residential, industrial, and agricultural activities are more influenced by land use than by bedrock lithology. Contaminants such as nitrate and chloride alter water quality, with nitrate being especially important due to its known health risks (Adimalla, 2020; Marghade et al., 2021a, 2021b; Subba Rao et al., 2020). Therefore, regular water quality monitoring is essential for sustainable water resource management.

## Conclusions

This study combines quantitative and qualitative methods to develop a conceptual model that captures the hydrogeochemical characteristics of a granite-based aquifer, which is representative of the geological settings commonly found in Korea. By focusing on a granite aquifer, the research provides a valuable reference for understanding the behavior and evolution of groundwater systems in similar domestic geological settings. In addition, the study area includes a mix of urban and green spaces, reflecting real-world land-use complexity in rapidly urbanizing environments. This makes the results especially relevant for groundwater quality assessments in urban regions with comparable geological and environmental conditions. Amid increasing challenges in groundwater management caused by climate change phenomena, including droughts and floods, this conceptual model offers critical insights into the current state of aquifer systems. It also helps identify potential contamination sources, thereby contributing to more proactive and adaptive groundwater management strategies. The conceptual model developed here, which captures both natural and anthropogenic influences on groundwater in a weathered granite aquifer, can therefore inform sustainable groundwater management in urbanizing regions. This highlights the relevance of the study in addressing both present and future environmental challenges.

Several limitations of this study should be acknowledged. Although additional sampling was performed in Yuseong-gu area, it did not cover both dry and wet seasons, and thus seasonal variability could not be fully evaluated. Future investigations should incorporate seasonal monitoring and explicitly distinguish between weathered and fractured aquifers to refine the conceptual model. Second, the geochemical simulations were conducted using representative sampling points that reflect the dominant water types in the study area. While this approach captures the major mineral reactions controlling groundwater chemistry, minor reactions could not be fully constrained. Additional isotopic or geochemical tracers will be required in future studies to better identify these subtle processes. Lastly, the groundwater flow path was primarily delineated based on topographic elevation and hydraulic head distribution, which provided a reliable regional-scale framework. However, the influence of subsurface fractures, particularly those that may locally alter flow directions in urban catchment (Lancia et al., 2020) could not be fully incorporated into the conceptual model. This limitation reflects the constraints of the available dataset and modeling approach.

## Data Availability

No datasets were generated or analysed during the current study.
